# Parents’ perspectives and roles in influencing 10–12-year-old children’s fruit and vegetable consumption: a qualitative study in Sweden

**DOI:** 10.1186/s12889-026-29852-2

**Published:** 2026-10-13

**Authors:** Hannah Helgegren, Anna Winkvist, Christel Larsson

**Affiliations:** 1 Department of Food and Nutrition, and Sport Science, University of Gothenburg, Box 300, Göteborg, 405 30 Sweden; 2 Department of Internal Medicine and Clinical Nutrition, The Sahlgrenska Academy, University of Gothenburg, Box 459, Göteborg, 405 30 Sweden

**Keywords:** Parents, Children, Fruit, Vegetables, Strategies, Perspectives, Food, Qualitative

## Abstract

**Background:**

Parents play a central role in shaping children’s health behaviours and dietary habits. Previous research has provided insight into parents’ feeding practices, especially regarding preschoolers. Less is known about how parents of 10–12-year-old children, whose fruit and vegetable (F&V) intake is particularly low relative to recommendations, perceive F&V and translate their intentions regarding F&V into everyday practices. This study explored parents’ perspectives on F&V, their reasons for serving them, and their experiences of influencing their children’s intake.

**Methods:**

Fifteen parents of 10–12-year-old children (12 mothers, three fathers) were purposively recruited from diverse socioeconomic backgrounds in southern Sweden and participated in individual interviews. Data were analysed using inductive thematic analysis.

**Results:**

Parents perceived F&V as beneficial, primarily due to vitamin content and immediate health effects. However, most were unfamiliar with recommendations and often considered relatively small amounts sufficient. Vegetables were primarily served at dinner, with less attention to other eating occasions, which may reduce opportunities to meet the recommended daily intake. Parents described a range of strategies to influence their children’s consumption, including mixing F&V into other foods, accommodating preferences, encouragement, and providing rules or benchmarks (e.g. 'Always two colours on the plate'). Some parents expressed concerns about sugar content, occasionally leading to unnecessary restriction of F&V. The analysis generated five themes regarding parents’ knowledge and beliefs, sources of information, preferences and resources, agency, and intentions. In addition, four parental roles were conceptualised to illustrate patterns in the intentions and strategies the parents used when influencing their 10–12-year-old children’s F&V consumption—*The Enforcer, The Accommodator, The Teacher, and The Role Model*—ranging from direct influence on consumption to more indirect and future-oriented approaches.

**Conclusions:**

The findings suggest potential mismatches between the participants’ perceptions of F&V, including their understanding of amounts, health benefits, and some facilitating feeding practices, and public health messages such as recommendations. More accessible guidance on why, but also on how, how much, and how often to serve F&V may support parents similar to the participants in the present study in promoting their children’s consumption.

**Supplementary Information:**

The online version contains supplementary material available at https://doi.org/10.1186/s12889-026-29852-2.

## Background

Parents influence their children’s food consumption both directly and indirectly, thereby having the potential to affect children’s health. This is done by purchasing, preparing, guiding, modelling, and responding to children’s food requests [1, 2]. Several studies have shown that parents’ ability to make fruit and vegetables (F&V) accessible to their children is influenced by family socioeconomic status, including parental education, occupational status, cultural background, and area of residence [3–5]. These circumstances contribute to variations in diet quality among children and reinforce inequalities in health behaviours and health outcomes across different backgrounds of the population [6, 7].

The WHO recommends an intake of at least 400 g of F&V each day for adults [8], whilst individual countries have approximately the same or higher recommendations, sometimes formulated as 4 cups or 5 portions a day [9]. An intake lower than the recommendations is associated with an increased risk of many diseases such as cardiovascular disease, type 2 diabetes and some forms of cancer [10–13]. Consuming F&V during childhood contributes to meeting the nutritional needs of the growing child [8]. As F&V are generally low-energy, nutrient-dense foods, they may also help reduce the risk of unhealthy weight gain and childhood obesity [14–17]. In addition, regular F&V consumption during childhood can help establish healthy eating and lifestyle habits that may persist through adolescence and into adulthood [18–20]. However, both children and adults in many parts of the world consume less F&V than recommended [21–23], which may contribute to an increased risk of adverse health outcomes.

Children in fifth grade (10–12 years old) are the group of Swedish children that consume the least amount of F&V. According to the latest national survey [24], only 5% of children in this age group meet the current Swedish recommendation of 500 g of F&V per day for adults and children aged over 10 years. The mean intake of F&V was 247 g (SD = 150 g) for girls and 200 g (SD = 151 g) for boys. This included what the children ate at home but also in school—where all Swedish schoolchildren are provided with a subsidised free hot lunch including vegetables. Yet, in a study by Olsson et al. [25] the subsidized lunch did not automatically give a substantial contribution to fourth-grade (9-11-year-old) children’s F&V intake. Since children consume most of their meals at home, they depend on their parents or caregivers providing meals including F&V to reach the recommendations for consumption.

Children’s consumption of F&V is strongly influenced by F&V availability and the child’s individual preferences—factors that are shaped by parental behaviours in multiple ways [26]. Correlations between children’s and their parents’ F&V intake have been seen to be at their weakest between the ages of 10 and14 year old but become stronger by the time the child is 17–18 years, and an association between children’s and parents’ F&V consumption is present until age 30 [27]. Previous research has partly explored how parents influence F&V intake [28, 29]. For younger children, strategies such as parental modelling, repeated exposure, and the use of non-food rewards (e.g., stickers) have been shown to enhance vegetable acceptance [30–34]. A recent systematic review and meta-analysis by Costa Lopes et al. [29] reported that verbal and visual encouragement to eat was associated with a small but significant increase in younger children’s F&V intake. The review also identified possible, albeit modest, associations between younger children’s involvement in food preparation, teachable moments, praise, and F&V intake. Among older children and adolescents, higher F&V consumption has been associated with home availability, parental modelling, and parental F&V intake [28], as well as negotiation around food choices [35, 36], whereas no significant associations have been reported for praise or verbal and visual encouragement [29]. However, since most previous research is based on quantitative observational studies, less is known about parents’ own thoughts and perspectives. Existing qualitative studies have primarily focused on parents’ experiences of feeding practices or barriers to a healthy diet or F&V consumption among younger children, with or without overweight/obesity [2, 37–39] and parents offering vegetables to preschool-aged children [40, 41]. A UK qualitative online survey of parents of 4-11-year-old children explored parents’ perspectives on F&V in relation to their children’s diet, and identified facilitators such as cooking skills, knowledge of recommendations, desire to increase F&V intake, and a wish for better health for their child [42]. Concerning parents of older children, one study examining caregivers of 10–14-year-olds found that children could influence their home food environment, that is, what F&V were purchased and available at home, particularly when requests made to the parents were polite, made in person, or added to the shopping list [43]. Further qualitative research addressing parents of older children, exploring experiences, rationale, and understanding of F&V public health messages and nutritional guidelines, remains limited. Considering the central role parents play in shaping children’s dietary intake and habits, it is important to increase understanding of their perspectives when feeding school-aged children.

Thus, the aim of this study was to explore parents’ perspectives on F&V, their rationale for serving them, and their experiences of influencing their 10–12-year-old children’s consumption.

## Participants and methods

### Study design

Semi-structured individual interviews were conducted with parents of children in fifth grade (10–12 years old). The interviews were analysed using an inductive thematic analysis adapted from Braun and Clarke [44].

### Ethical considerations

The study was approved by the Regional Ethical Review Board at the University of Gothenburg before data collection commenced (Dnr 675 − 17). Participants provided verbal and written consent before data collection began. They were informed that participation was voluntary and that they could withdraw at any time without giving a reason. The collected data were analysed and stored in a way that ensured anonymity.

### Participants and recruitment

A purposive sampling method was used when recruiting 15 families. This was done jointly for this study and a connected interview study concerning children’s perspectives on F&V [45]. Recruitment was conducted through seven schools in two geographical locations in southern Sweden: a larger city with a multicultural population and a smaller village with a more ethnically homogeneous population. The aim was to recruit a diverse sample of families with regard to cultural background, area of residence, parental education, the child’s gender, and the child’s perceived interest in eating F&V. By seeking variation across these characteristics, we aimed to capture a broad range of experiences and perspectives related to F&V within families. The participating schools were located in areas of low, medium, or high socioeconomic status (SES), classified according to official school-level statistics on parental education. Schools in which 52–62% of parents had more than 12 years of education, corresponding to the city-wide average, were classified as medium-SES. Schools in which fewer than 30% of parents had more than 12 years of education were classified as low-SES, whereas schools in which more than 75% of parents had more than 12 years of education were classified as high-SES [46]. This classification based on parental education also corresponded with the proportion of families in which both parents were born outside Sweden, ranging from 90 to 93% in low-SES areas to 39% in medium-SES areas and 19–20% in high-SES areas. In the village schools, 49% and 70% of parents had more than 12 years of education, while 25% and 8% of families, respectively, had two parents born outside Sweden [47]. For comparison, national statistics from the same year showed that 58% of parents had more than 12 years of education, and that 25% of children had two parents born outside Sweden [48]. Teachers in each school assisted with recruitment by identifying potentially eligible children in fifth grade (10–12 years old) and distributing invitation letters to their families. Teachers were asked to recruit families with varied backgrounds and to include both children who were perceived as readily accepting F&V and children who rarely or never ate F&V during school lunches. Once an invited family expressed interest in participating, the parent(s) contacted the study contact person and received further information about the study by telephone. If they agreed to participate, the parent(s) or legal guardian(s) decided whether one or, where applicable, two adults from the family would take part in the study. The inclusion criterion was being the parent or legal guardian of a child in fifth grade, and the exclusion criterion was not being able to provide informed consent. When 15 families had been recruited and interviewed, and after reviewing notes from the interviews, the research team judged that the data were sufficiently rich and varied to address the study aim, and no further invitations were distributed.

Prior to the start of the interviews, all participants completed a study-specific background questionnaire. The questionnaire included a free-text section in which participants were asked to describe the child’s household composition, including individuals living with the child across one or more households in cases of shared residence, and their relationship to the child (e.g., mother, father, step-parent; see Appendix A).

### Data collection

Individual semi-structured interviews were conducted with each participant. The location (home, a children’s hospital or the child’s school) was chosen by each participant to facilitate recruitment and participation from families with varying socioeconomic circumstances [49]. The interviews were digitally recorded using MP3 devices and subsequently transcribed verbatim. An interview guide was developed in advance and applied consistently across all interviews, complemented by probing questions and prompts to encourage elaboration. The questions were designed to elicit participants’ thoughts, experiences, perceived obstacles, strategies, and knowledge regarding F&V, see Appendix B for an English translation of the interview guide. At the end of each interview, participants’ awareness of current F&V recommendations and their satisfaction with their family’s consumption were explored. This sequencing was deliberate to avoid influencing earlier discussions about experiences and rationale for serving F&V. The first two interviews were initially considered pilots; however, as no revisions of the interview guide were necessary, they were incorporated into the dataset. A set of colour photographs depicting 28 different vegetables and 25 different fruits and berries was used at the beginning of each interview to help participants focus on the same phenomenon (F&V). The material was then visible throughout the interviews and was used as an aid when participants lacked specific names or words to describe F&V. During the interviews and subsequent analysis, the definition of what constitutes a vegetable or fruit and berry was guided by common usage rather than strict botanical classifications. Participants were informed that potatoes were not classified as F&V in this study.

### Data analysis

An inductive thematic analysis, adapted from Braun and Clarke [44], was conducted. The initial coding was carried out manually using pen and paper, with NVivo 14 software (Lumivero) employed for data storage and later coding management. The process began with repeated readings of the transcribed interviews, maintaining openness to the content. Audio files and interview memos (written by HH immediately after each interview) were consulted as needed for clarification. Throughout the analysis, fruit and vegetables were considered both separately and collectively. Parents’ views on fruit and vegetables were analysed as distinct phenomena, as well as jointly as a single food group. Text segments relevant to the study aim were marked with preliminary codes that were developed inductively during the analysis. The coding focused primarily on manifest content but also incorporated a limited amount of latent content, such as underlying values or views inferred from participants’ statements and strong emotional expressions. Longer meaning units were condensed when necessary, and each interview was summarised to enable reflection on it as a whole. Segments were then compared across interviews to identify similarities and contrasts. Codes and text segments were grouped and refined through an iterative process, moving between transcripts and emerging interpretations. The final steps involved organising the structure, assigning descriptive names to themes and sub-themes, and writing up the results. After the thematic analysis, some findings were further conceptualised to illustrate patterns that were discerned from parts of the data. The conceptualisation was developed following completion of the thematic analysis and was not an a priori component of the study design.

The study was informed by a social constructivist orientation [50], recognising that parents’ understandings and practices around fruit and vegetable provision are shaped through social interaction and context. The pre-understanding of the researcher primarily responsible for the analysis (HH), who conducted the interviews and initial coding, was consciously drawn upon throughout the analytic process. This included extensive experience as a registered clinical dietitian working with families in diverse socioeconomic and cultural contexts. Conscious reflexivity was actively maintained during both the interviews and the later analysis, with ongoing reflection on positionality and pre-understanding. Pre-understanding was regarded as both a resource and a potential influence on interpretation. Throughout the analytic process, efforts were therefore made to draw upon professional experience while remaining attentive to perspectives and interpretations that differed from prior expectations. The second and third authors (AW and CL), both senior nutrition researchers with previous qualitative research experience, contributed throughout the analysis by reflecting on codes, themes, and categories, thereby supporting trustworthiness. The analysis and its results were also discussed with peers. The interviewer (HH) and the rest of the research team had no previous relationship with, or knowledge of, any of the participants. However, the schoolteachers who distributed the study invitations had an established teacher-student relationship with the children and were, to varying degrees, familiar with the parents. The teachers were not involved in the actual interviews or the analysis.

## Results

In total, 15 parents (12 mothers and three fathers) participated in individual interviews conducted between April 2018 and January 2019. Among the participants, eight opted for interviews at home, six at an office within the local children’s hospital, and one at the child’s school. Interviews lasted between 46 and 80 min (mean 57 min). Parental background characteristics are summarised in Table 1. All participants identified themselves as either ‘mother’ or ‘father’ and were therefore referred to accordingly during the interviews and subsequent analysis. All interviews were conducted in Swedish. Nine participants did not have Swedish as their first language, although all considered their Swedish language proficiency sufficient to participate in an interview without an interpreter. Their proficiency in Swedish varied, ranging from fluent speakers to participants with more limited proficiency who were nevertheless able to provide detailed accounts of their experiences. Some participants occasionally used simpler vocabulary or sentence structures and, at times, referred to visual aids depicting fruit and vegetables or searched for specific food items on their smartphones.

**Table 1:** Background characteristics of 15 parents participating in interviews

Women (men)	12 (3)
Born in Sweden	6
Born in other European countries*	6
Born outside Europe*	3
Education ≤ 12 years	5
Education > 12 years	10
Recruited through school in lower SES area in city Recruited through school in medium SES area in city Recruited through school in higher SES area in city Recruited through schools in smaller village area	3 5 3 4

* Participants that were not born in Sweden originated from: Bosnia, Denmark, Egypt, Germany, Norway, Pakistan and Syria

The parents were interviewed about their children in 5th grade, who constituted the primary focus of the study. Among these children, eight were girls and seven were boys, ranging in age from 10 years and 11 months to 12 years and 2 months. The children attended schools in areas with varying socioeconomic status (SES), with three attending schools in low-SES city areas, five in medium-SES city areas, four in medium-SES village areas and three in high-SES city areas. The total number of children in the participants’ families ranged from one to five. Most families had two (*n* = 8) or three (*n* = 4) children, whereas one family each had one, four, and five children. Twelve of the families had children who were born in Sweden. No family chose to include more than one parent in the interviews, meaning that each family was represented by only one interviewee. Among the participating parents, current labour market status was working (*n* = 10), student (*n* = 2), on parental leave (*n* = 1), on long-term sick leave (*n* = 1), and retired (*n* = 1). None of the participating parents were officially unemployed, although one parent’s partner was. The parents’ current spouses/co-parents contributed to each family’s socioeconomic status, generally having a lower or similar level of education as the participating parent (*n* = 2 ≤ 9 years of education). The participating parents’ partners were born in Sweden (*n* = 6), other European countries (*n* = 4), and countries outside Europe (*n* = 4). Household income among participants’ families ranged from approximately €20,000 to €80,000 or more per year. Information about parents’ and children’s weight status were not collected during data gathering and was not a part of the purposive sampling strategy. Nonetheless, both normal-weight and overweight parents and children were represented within the sample.

The analysis produced five themes with associated sub-themes, as shown in Table 2 and described in the following sections. In addition, a conceptualisation of four parental roles was developed from parts of several themes, illustrating parents’ intentions and strategies when influencing their 10–12-year-old children’s F&V consumption.

**Table 2:** Results from individual interviews on parents’ perspectives regarding fruit and vegetables and their experiences of influencing their children’s consumption, presented as themes and associated sub-themes

Theme	Sub-Themes
Parental knowledge and beliefs	o Current, not future health o Sugar-related hesitation & not real fruit o Just a little is enough o To buy or not to buy organic o The apple doesn’t fall far from the tree
Sources of information	o Peer guidance and community o Absent or disobliging authority advice o Skewed marketing o Accessing various media o Who to trust?
Family preferences and resources	o Parents set the scene o Children bring fuel or friction o Balancing family’s preferences o Cultural transitions o On a Budget
Parental Agency	o Overcoming everyday hurdles o Responsibility and rules o Intentional restriction o Knowing what to do if needed o Satisfied with current consumption
Parental intentions	o Providing nutrients o Providing access o Providing knowledge & skills o Providing models

### Theme: parental knowledge and beliefs

Parents’ decisions around F&V were described as a complex interplay of health-related beliefs, nutritional knowledge, and personal values. Their reasoning reflected both informed choices and uncertainties, revealing how perceptions of health, sugar, organic farming, and dietary norms could influence everyday practices.

#### Current, not future health

Parents cited vitamin and mineral content as the main reason for children to consume F&V, while a few also mentioned fibres and a perceived ‘lack of additives’ as positive attributes. Associations of F&V with general health benefits, such as growth, energy, and immune defence against infections were common, but a limited number of parents also offered more specific claims—such as preventing constipation, supporting gut bacteria, or enhancing their child’s academic performance. *‘…the body needs vitamins*,* minerals and this power that we need. …I consider vegetables as the best source. But also*,* children often have stomach-ache*,* and sometimes constipation*,* so it’s good if you can cleanse. We usually say that plums are really good*,* pears are really good*,* and that if you have diarrhoea banana is good. But mostly it’s just vitamins*,* they are rich*,* providing vitamins.’ —Mother*,* J.* Participants rarely included long-term health considerations in their reasoning and only one parent connected the desire for the child to maintain a healthy diet and eat F&V with concerns about the child’s susceptibility to diabetes and obesity (which were common among relatives). When comparing F&V with other foods, snacks, or sweets, many parents concluded that F&V were the healthier choice. *’I think it’s better than eating sweets and other stuff*,* like potato chips and similar… You don’t get completely full when you only eat a fruit… (but) you still get a small sense of a reward’.—Mother*,* A.* Although most viewed F&V as essential, one parent questioned their necessity, suggesting that vitamin supplements could serve as an adequate alternative and that F&V was optional. *‘The thing about fruit and vegetables*,* I don’t think it’s them that keeps us healthy*,* (and) makes us not sick. I don’t think it has anything to do with that at all… I mean*,* they (children and adults not eating F&V) are not malnourished*,* but they have a deficiency of vitamin A and B and C and all that. You go to the doctor and you get a tablet for it’.—Mother*,* M.*

When reflecting on their own diets, parents cited similar motivations to those mentioned for their children, with the addition of weight management and possible cancer prevention. Notably, such long-term health concerns were not mentioned in relation to children by the majority of participating parents. A few parents reported forcing themselves to eat specific F&V they clearly disliked—such as broccoli or avocado—because these were perceived as particularly healthy. *‘Broccoli*,* I force myself to eat it… you have to. I know it’s good for your health and for cancer’. —Mother*,* L.*

#### Sugar-related hesitation and not real fruit

A concern that fruit consumption might need to be restricted due to its sugar content was expressed by more than a few participants. *‘I want to think it’s healthy (fruit)*,* but I think that it also contains quite a lot of sugar’. —Mother*,* N.* Two parents reported having received dietary advice from personal trainers, who had suggested that the sugars found in fruit were harmful and should be limited or excluded. *‘What I learned from my PT is that you should not eat fruit because it increases your blood sugar… So*,* I think it’s a bit problematic with fruit… I still have the advice from my PT in the back of my mind*,* concerning this*,* concerning blood sugar.’ —Mother*,* C.* Consequently, some parents reported avoiding very ripe fruit or refraining from offering canned sweetcorn. One parent did not want to make a dinner perceived as ‘unhealthy’ (pizza) even more ‘unhealthy’ by offering a white cabbage salad, a side dish commonly served with takeaway pizza in Sweden, because of concerns that the seasoning might contain added sugar. *‘Pizza salad is rarely very healthy*,* I feel that if I occasionally serve pizza I don’t need to make it worse than it is (by serving the salad)… That kind of (seasoning) mix often contains sugar.’ — Mother*,* A.*

It was common to associate fruit juice with sugar, and several gave voice to the belief that store-bought juice often contained added sugar, more sugar, unknown additives or artificial ingredients. Some parents suggested that homemade fruit juice is a better option than store-bought juice and could be more comparable to eating whole fruit, but many families had rules or routines that limited juice consumption during weekdays, considering fruit juice more of a weekend luxury. *‘If it’s homemade (juice) it’s fruit*,* but if it’s bought in the store… well I don’t know how real it is*,* but I have heard that [brand name] is better than the other artificial ones*,* like it’s not real fruit in them’. — Mother*,* J*.

#### Just a little is enough

None of the parents reported knowing the exact national recommended daily intake of F&V, although four gave a correct answer, 500 g, when asked to guess. Some underestimated the recommendation (suggesting approximately 250 g) but later elaborated by listing examples of F&V that could collectively amount to around 500 g. One parent mentioned ‘Five different things each day’ while another referred to the phrase ‘Five a day.’ *‘I’ve read it (the recommendation) once*,* but I don’t remember so well what I read*,* so I’ll guess… I think it’s quite little*,* maybe 250 g or something? …Once upon a time you said “five a day” but I don’t know if you do that anymore. But that’s my guess. But now I’m uncertain if that maybe is only fruit? Maybe it’s not vegetables included… I suspect that vegetables are included.’—Mother*,* D.* The remaining participants’ guesses ranged from 100 to 1000 g, with only one exceeding the actual recommendation and the rest falling below it. *‘Maybe 100 grams a day. I don’t know’… I mean*,* it’s not often you see someone eating 2–3 apples a day or*,* eh*,* each snack is an apple or a banana or something. I don’t manage to eat fruit daily. It’s really hard’. — Mother*,* M.* Most estimates aligned with the average intake among Swedish adults, typically between 300 and 350 g per day. Another initially assumed that the recommendation applied to the entire family rather than to individuals.

#### To buy or not to buy organic

Concerns about pesticides in F&V were raised spontaneously by several parents, who viewed this as a complicating factor when deciding what to buy and serve. *’I wouldn’t eat grapes that are not organic… When I have read that something is heavily sprayed (with pesticides) I buy organic*,* if the price difference is not gigantic. …You are a bit scared of what all these pesticides do… for the children of course*,* but also for me*,* but the children are always the priority’.—Mother*,* D.* Many parents described organic produce as expensive and therefore not a priority, although some preferred it for specific items. Conflicting information about the safety and benefits of organic options contributed to uncertainty. One parent compared fresh produce with packaged snacks, noting that ingredients in snacks are clearly listed, whereas pesticide content in F&V remains unknown. *’It’s really difficult… It would have been good if it said how much pesticides there is*,* so you know what you are buying exactly. So*,* when you buy something*,* like potato chips*,* you know what it contains. What oil. Well*,* I don’t want palm oil*,* so I take sunflower seed oil. It’s like that with vegetables*,* you choose. This has pesticides*,* I’m a bit more (wanting to get) organic. But I’m confused’. —Mother*,* K.*

Another parent dismissed organic produce entirely as a costly marketing strategy. *‘I don’t believe in organic. I think it’s crazy. It’s just cheating. I think they are hoaxing people from their money. … We can be sitting at work talking about organic apples and I will say “but wait, all apples come from a tree. What is the difference?” It’s just the packaging and they will put organic sticker or kravmärkt (a controlled organic label) on it. I don’t understand how people can agree to it. I myself have compared it, an organic apple and a normal apple, and they stay good about the same amount of time.’ — Mother, M.*

#### The apple doesn’t fall far from the tree

When considering families in general—not only their own—parents attributed variations in children’s F&V intake primarily to parental influence, noting that low consumption among adults often translated into limited offerings for children. *‘I think it’s the parents. How you have things at home*,* how we serve*,* force*,* choose for yourself… You might not think differently because you are used to it*,* this is how we have it (at home) and then you believe that it shall be like that.’ —Mother*,* F.* Some participants believed that a lack of interest, knowledge, or confidence in preparing F&V could hinder other parents’ encouragement or provision of F&V. *‘It’s the parent. Totally the parents…. There is no child that doesn’t like a carrot. It’s the parents that don’t dare to try it or serve it. …No child will sit hungry and just (not eat). They will eat*,* and if they don’t*,* well 2 hours later they are hungry and they will eat it… I think it’s what the parents themselves are feeling safe with… they might not have seen them (different F&V varieties)*,* probably have not tasted them. So sure*,* they will go shopping and pick up cucumber and tomatoes because they feel safe with (only) those’. —Father*,* H*.

### Theme: sources of information

Parents described navigating a wide range of sources of F&V information, sometimes encountering conflicting advice and describing uncertainty or scepticism when trying to make informed decisions.

#### Peer guidance and community

All participants referred to various sources of information about F&V. Many had acquired knowledge about eating and preparing F&V during childhood. *‘Everybody has grown up with the food pyramid in some way I think… It was probably my mom (who taught me about F&V)*,* yes my mom I would say. And then school. And me doing sports when I was younger… to perform you need to eat properly’.—Mother*,* N.* Others had received more recent advice concerning nutritional properties and health benefits from sports clubs and personal trainers. One interviewee shared that her parents often provided her with advice concerning her children. They had recommended serving apples to protect her children’s teeth, watermelon during the summer for hydration, and tomatoes to benefit the blood. A colleague at work had advised the same participant to serve bell peppers to improve the child’s general well-being. Other participants likewise mentioned receiving guidance from colleagues at work. *‘I have a colleague; she says that children should eat fruit before they have their snack (sandwich or porridge). She also tells me that the children must eat fruit before they go to bed. I have a few acquaintances with other opinions*,* but when (the colleague) said this to me I thought that yes*,* I must try.’ — Mother*,* K*.

#### Absent or disobliging authority advice

Several parents recalled receiving helpful dietary advice about F&V from paediatric health nurses during their child’s toddler years and early childhood check-ups. However, as children grew older, such guidance from official sources diminished. Advice from other institutions, such as sport clubs their children attended was sometimes received, but not always welcomed by the parents. *‘The (children’s) football club is always giving advice about what you should and shouldn’t do*,* and they want us parents to attend lectures about what the kids can and can’t eat*,* but I’ve refused to go… I get really provoked… “you should not eat potato chips*,* you should eat a carrot instead”.’ — Mother*,* B.* While schools were seen to promote F&V, this messaging was directed at children rather than parents, which some parents appreciated. *’It’s better that teachers and the school nurse talk to the children about healthy things*,* that you should eat more fruit and vegetables than other (things)’. —Mother J.*

A few participants mentioned encountering advice from government or health agencies, though this was sometimes met with scepticism due to confusion about the source or intent of the information. One participant referred to ‘government food guidelines’ in a way that appeared to reflect a misunderstanding of both the source and the content of the message, recalling a 1976 commercial produced by a bread industry lobby organisation stating that “The National Board of Health wants you to eat 6–8 slices of bread each day”. Although this was never an official dietary recommendation, the message has remained in the minds of many Swedes as a perceived dietary guideline. *‘ (Advice) that are standardised and given the same to all… it’s the same as when the County administrative board (means National board of health) invented the recommendation of 3 slices of bread per day. You see? That all should eat 3 slices… I think it’s silly to provide these (general advice)’ —Father*,* P.*

#### Skewed marketing

Several parents reflected on the influence of media and advertising, noting that F&V are rarely promoted compared with more processed or unhealthy foods. *‘Its not a lot of commercials for vegetables on the TV or in media*,* it’s more about milk products… and happy pigs. And “this week’s shopping item” (promoted product on sale at the supermarket) is usually never fruit or vegetables’. — Mother*,* N*. One parent observed that fast-food chains attempt to appear health-conscious by including apple slices or carrots, although these options were perceived as rarely consumed when served alongside fast food. Colourful packaging and strategic placement of unhealthy products in supermarkets were seen as particularly appealing to children, making it difficult for parents to prioritise healthier choices such as F&V during shopping. *’…the unhealthiest (foods) has the brightest*,* happiest and most colourful packaging*,* it’s understandable that it’s hard to be in the food store with kids that want everything like that… and then you might think that*,* well this is made for kids so it must be ok’. —Mother*,* D.*

#### Accessing various media

Parents described a range of information sources related to F&V, including tv shows, search engines, podcasts, radio programs, Instagram, and other social media platforms. *‘I like watching shows about food*,* like vegetables or fruit*,* and always when there is a disease*,* for instance if you have a cold*,* I go online and try (to find) what fruit is best against a cold. So now I know that kiwi is really good and has lot of vitamin C’. —Mother*,* L.* Content was accessed both intentionally and incidentally. Several participants considered internet searches effective, especially when results appeared to be supported by scientific studies or university affiliations. One parent valued learning about unfamiliar F&V varieties through online resources. *‘In Sweden you eat a lot of broccoli*,* so when we came here (from Syria) we used Google to ask why broccoli? And then*,* aha*,* okay*,* so we need to eat broccoli.’. — Mother*,* L.* Parents with a particular interest in food and health also mentioned reading books and listening to audiobooks and podcasts featuring healthy foods, such as F&V, sometimes together with their children. *’I buy a lot of books… We found one called “Healthy family in 30 days” …you make it accessible for the children*,* look and read together.’ —Mother*,* C.*

#### Who to trust?

Several parents expressed uncertainty about which sources of information to trust when messages were conflicting. The messages could originate from various channels, including friends, magazines, social media, and personal trainers, and often involved claims related to F&V and health, dietary practices, or the legitimacy of organic farming. *‘On TV*,* and then*,* well I haven’t read it*,* but we talk at work and someone else has read that it’s not a big difference between organic and non-organic*,* so it’s a bit like: who to trust?’ — Mother*,* K.* Some participants highlighted the role of mainstream media in circulating messages that may discourage the consumption of F&V. For instance, it was described that media was spreading health warnings about specific foods or suggesting that cucumbers and green salads are nutritionally insignificant and therefore pointless to consume. Such messages were seen to foster unease around food in general, and F&V in particular. *‘Saying some kinds of fruit or vegetables are giving people cancer. I’ve heard it and I react strongly. Mushrooms*,* and not long ago it was green salad leaf*,* it was the same. And you kind of wonder “what are you trying to do with all that?” It’s the news and the magazines.‘ — Mother*,* M.* The uncertainty around the credibility of these warnings—and suspicions of underlying agendas—occasionally made it more challenging for some parents to make informed decisions about purchasing and serving F&V, and sometimes made the parents more unwilling to listen to dietary advice.

### Theme: family preferences and resources

Family dynamics, preferences, and available resources were all described as affecting the consumption of F&V within the home. Family food practices were complex, and individual needs and values had to be balanced within shared routines.

#### Parents set the scene

All parents reflected on their own preferences regarding F&V, including favoured varieties, tastes, textures, and serving styles. *‘I don’t like fruit very much. I force myself to eat it*,* I try. I always choose (those varieties) that are hard*,* not soft. And that has more acidity.’ —Mother*,* L*. These preferences sometimes influenced which F&V parents were most willing to prepare and offer to their families. However, even when parents disliked particular F&V, they often made deliberate efforts to include them if other family members enjoyed them or requested them. *’(Okra/Lady fingers). …my oldest children love it but I don’t like it. When you eat it it’s the same as mushrooms*,* a bit slimy. That’s why I don’t like it… It’s not on my “list”. But I always make it when my children are at my place.’ —Father*,* P.* One participant reflected on his own consumption choices, weighing the caloric content of F&V against the enjoyment he derived from alternative foods and beverages. *‘It’s a lot of energy for very little flavour. If you consider a banana*,* I would rather have a beer. It’s basically the same energy content… (laughing) but one (the beer) is really good and the other is bland and tasteless.’ —Father*,* E.* This suggests that parents may apply different criteria when considering F&V for themselves compared with when choosing foods for their children.

#### Children bring fuel or friction

The participants acknowledged that each child has their own preferences for F&V, which significantly affect what they are willing to eat. *’She eats bell peppers*,* and they should be yellow*,* red or orange*,* not green and not served cooked in any way.’—Mother*,* C.* Parents also noted that these preferences may change over time or be influenced by varieties and serving styles encountered at school or at friends’ homes. *‘She (the child) didn’t want to eat avocado at all. Then she had it at a friend’s house*,* and she came home and asked -why don’t we ever have avocado? (laughing) So now we eat avocados*,* but (we serve it) in the same way as they do at her friend Jennie’s home’. —Mother*,* A.* Some parents pointed to specific sensory features of F&V that deter children—such as bitter flavours, unappealing appearance, or off-putting textures like overly soft or hard. Others emphasised that a child’s hunger level is crucial; if the child is not hungry enough, they will not eat vegetables. Serving style was frequently mentioned, with many parents aware of how their children preferred F&V to be served—some mostly raw, others cooked, or fruits cut into bite-sized pieces or presented in a playful way. *‘Our child wants it served like for a (younger) kid*,* a big platter and every vegetable by itself (not touching)*,* no dressing… Then sit and pick them (individually)*,* not putting it on his plate*,* no*,* he wants the big plate in the middle (of the table)*,* and everyone can pick (vegetables)*,* preferably with their fingers’. — Mother*,* B*. Children who expressed a liking for particular F&V created a demand for it, which parents could potentially meet, depending on factors such as family economy, time, season, and willingness to accommodate the child’s preferences.

#### Balancing family’s preferences

Balancing different preferences within the family was described as a challenge. The parents often had to decide which child’s F&V preference to prioritise when dietary habits differed. *‘They like very different things. [Childs name] for example*,* he loves tomatoes*,* and his sibling doesn’t like tomato*,* it’s the vegetable she likes the least. She loves mushrooms*,* but he doesn’t… it’s sure they have different preferences.’ — Mother*,* B*. One parent described how one child insisted on knowing exactly what was being served, while the sibling would only eat vegetables ‘hidden’ in the meal. While many parents tried to accommodate each child’s preferences, those with multiple children noted the difficulty of keeping track of who was currently eating—or avoiding—specific F&V. Some parents observed that children’s acceptance of F&V varied depending on which adult was present, one noting that the child complained when the mother served food but ate without protest when the father was serving. Differences in food culture between parents also influenced F&V choices; for example, one parent preferred canned or cooked vegetables, reflecting childhood habits, while the other favoured raw produce. *‘I like vegetables*,* but it’s hard to get my husband to eat vegetables… At dinner he can be a bit mad at me*,* because I have put a lot of vegetables on the table*,* and he doesn’t think it’s proper food… he eats English style’. — Mother*,* A*. Parents of children living in shared custody arrangements due to divorce could describe noticeable differences in exposure, with one household serving F&V daily and the other rarely, if at all. *‘When [the child’s father] lived by himself (before meeting a new partner)*,* it was nothing. No vegetables or fruit at all. There was a contrast (between the two home environments). …There was nothing at all.’—Mother*,* F.*

#### Cultural transitions

Parents with migration backgrounds described some contrasts in food cultures across national contexts. *’It’s a bit different*,* here (in Sweden) you always eat a hot lunch at school*,* you don’t do that in (parent’s home country)’.—Mother*,* A.* The parents found themselves navigating the F&V they were familiar with and those their children had become accustomed to in Swedish food culture. *‘The problem with vegetables here in Sweden or Europe is that… they taste nothing! Just cooking them and eating them only cooked. In Egypt we call that hospital food. You see? … The difference between hospital food and home food is that home they can afford spices*,* and when there are 1 million spices in the world… the Swedes are very bad with spices. It’s a fact’.—Father*,* P.* Some parents expressed uncertainty about the varieties available in Sweden, which sometimes deterred them from purchasing certain types of F&V. Participants also commented on cultural differences such as whether fruit is incorporated into hot dishes and preferences for serving vegetables either cooked, canned or raw. Within families, diverse views and preferences often coexisted—particularly when parents came from one cultural background and the children, raised in Sweden, developed different preferences. One parent referred to this phenomenon as ‘the school effect’, voicing frustration that their child now preferred other apples, bland non-spicey vegetables, and Swedish cheese. *’(In my food culture) we never eat green apples. We think it’s tart and sour. But they (the children) eat it. It’s their best one. They think it’s typical Swedish’. —Father*,* P.*

#### On a budget

Parents frequently cited household finances and the cost of F&V as key factors shaping what was available at home. Price influenced not only the quantity but also the types of F&V purchased. *‘For a while*,* my daughter was really into pomegranates*,* and it happened to be when they were crazy expensive*,* so I just thought*,* no*,* I’m not going to (buy them).’ —Mother*,* G.* Parents from both lower-income, and more affluent households, acknowledged that economic constraints may affect many families’ ability to buy F&V. *‘…economy matters… I think those with good economy eats more fruit and vegetables’. —Mother*,* L.* One parent from a lower-income background remarked that while some families could afford to send fruit salad to school as a snack, this was not financially feasible for her. A few participants reported that when budgets were tight, they reduced their own intake of F&V to prioritise their children’s consumption. *‘I don’t have a lot of money*,* so I prioritize my children. It’s not that I don’t want to eat more myself*,* it’s just that I make sure they get it also. It’s a matter of prioritising. If I notice it will cost a lot of money I will not buy it*,* economy plays a large role when I buy food. So if I see pineapple on sale I buy pineapple*,* if there is carrots… Well*,* I always buy carrots’. —Mother*,* A*. In contrast, one parent argued that although others complain about the cost of F&V, prices are relative, and meat is considerably more expensive—making F&V a more economical choice.

### Theme: parental agency

Rather than acting as passive providers of F&V, most parents navigated everyday challenges, set expectations, or made decisions based on their values and perceived responsibilities, demonstrating a sense of ownership. They were in general mostly satisfied with their children’s current intake of F&V but felt they had the agency to make changes if necessary.

#### Overcoming everyday hurdles

While parents recognised several barriers to their children’s consumption of F&V, they also described actively working to overcome these challenges. Rather than viewing obstacles as insurmountable, parents reported using a range of strategies to support F&V consumption, particularly within the home environment. For example, some parents described adapting their purchasing and food preparation practices to manage financial constraints while maintaining children’s access to F&V: *‘We try to buy what is in season. Now it’s autumn*,* so there are lots of apples. We also pick berries from our garden*,* and we go to (a farm) and pick raspberries ourselves and freeze them. We originally started freezing them for economic reasons. Then we add the frozen berries to the morning porridge. I try to buy things when they are on sale or in season.’—Mother*,* C.* Table 3 presents examples of strategies parents used to navigate such challenges. 

**Table 3:** Examples of strategies used by participating parents to address perceived barriers to their children’s fruit and vegetable (F&V) consumption

Perceived barrier	Strategies used by parents
‘F&V is expensive’	Shop at budget-friendly outlets; Buy smaller quantities or frozen; Prioritise serving F&V to children over adults.
‘Vegetables can taste bitter’	Roast vegetables in oven to enhance sweetness.
‘Children dislike the F&V that is served’	Temporarily stop offering disliked varieties; Provide alternative F&V options; Make F&V into playful shapes and figures; Use subscription of Meal Kit and refer to the set menu.
‘Children refuse F&V at dinner’	Offer F&V as snacks; Serve slices and pieces immediately after school when the children are hungriest, while dinner is being prepared.
‘Fruit contains sugar’	Serve fruit in moderation; Avoid very ripe fruit.

#### Responsibility and rules

Many parents expressed a strong sense of responsibility for their children’s consumption of F&V. *‘As long as they live in my house it means they are my responsibility. As a Muslim I’m responsible until they move from home*,* …you cannot throw someone into the ocean and tell him ‘You must swim’ when you have not taught him how to swim. But teaching him to swim means you doing your duty as a parent. Or if he is in the water and doesn’t want to swim. it’s up to him. …but I see this from a religious point of view… it’s my job to give him good health. And a good health means that he will eat a varied diet ‘ —Father*,* P.*

In many cases the feeling of being responsible was accompanied by clear expectations, aspirational practices, or explicit rules around how much F&V should be consumed or included on the child’s plate at dinner (see examples in Table 4). Similar rules or informal benchmarks could be applied to fruit, especially since it was often eaten as a snack without the parent being present. Some parents set rules on quantity, either a minimum or a maximum amount a day. In contrast, a few parents rejected the idea of rules altogether, preferring to let the child decide freely what and how much to eat. As one participant explained: *‘If I buy 2 kg of grapes and [the child] eats it all*,* he can. And if he doesn’t want them*,* then he doesn’t want them.’* — *Mother*,* M.*

**Table 4:** Examples of family rules or expectations that the parents of 10–12-year-old children described during individual interviews regarding their children’s consumption of fruit and vegetables (F&V)

Expressed rule or expectation	Parent/child	Parental practice
’Always one vegetable every dinner’	Parent serves	Providing
‘Always two colours of vegetables on the dinner plate’	Child puts on the plate	Providing
’No meal without vegetables’	Parent serves	Providing
’Always try one bite’	Child consumes	Encourage/Pressure
‘Must eat and finish what F&V has been served by parent’	Child consumes	Pressure
‘One fruit a day is minimum to eat’	Child consumes	Pressure
‘Only one piece of every kind of fruit is allowed per day’	Child consumes	Restriction
‘Don’t eat "luxury fruit"/berries without asking first’	Child consumes	Restriction

#### Intentional restriction

Several parents reported feeling a need to limit their children’s consumption of F&V, particularly fruit (see example of restriction in Table 4). This was mostly done using overt restriction, where the child was aware of actively being restricted. For some parents, the restriction was due to a perceived risk of overeating, where one or more children in the family consumed such large quantities of F&V that the parents were afraid it would interfere with their intake of other important food groups. One parent described this situation, noting that the child could ‘chew all day’ and that fruit was deliberately and overtly restricted using rules for consumption, but also more covertly restricted by being concealed in the house to make access more difficult. *‘…we need to put a stop. One of each kind is ok. So it can be one apple*,* one banana*,* one small citrus fruit*,* if its season. Otherwise pear*,* apple*,* banana. We usually have some in a bowl and some in the bottom of the fridge. But now when it’s season for citrus we have a secret stash*,* we can only put 2 at a time in the refrigerator. I don’t even know where the rest are right now’. — Father*,* E.* Another parent mentioned limiting specific varieties of F&V during early childhood, having been advised that kiwi contained ‘too much iron’ and bananas ‘too many carbohydrates’, but the child being too young to understand that there was a restriction. A few parents expressed concern about the sugar content in fruit or dental health, stating that they tried to restrict fruit intake to allow the child’s teeth to rest between meals or to reduce perceived sugar consumption. *‘You need to let your stomach and teeth rest … even if fruit and vegetables are healthy*,* you shouldn’t be eating them all the time because there’s fruit sugar in them*,* and that’s not good for your teeth.’—Mother*,* O.* Others limited access to expensive or exotic fruits, reserving them for special occasions or ensuring they were shared fairly among family members.

#### Knowing what to do if needed

Most parents believed they could increase their children’s consumption of F&V if they wanted or needed to, and they could describe how they would do it, thereby expressing a sense of control and confidence in their ability to influence intake. *‘I would probably have made them (F&V) look nice on the plate*,* yes*,* and maybe a tasty sauce to dip it in. You also eat with your eyes so it must look good. And I would make sure to serve them (F&V) at every meal and make sure we always have varieties that I know the children like at home. …Maybe put more fruit and vegetables on the plate. It happens that we forget it sometimes at dinner and have no vegetables at all. We could get better at having it at home*,* preparing it and putting it on the plate’. —Mother*,* O.* Common strategies parents said they would use if they wanted to increase their child’s F&V intake included serving vegetables more frequently, accommodating children’s preferences more often, and making F&V more accessible, such as cutting F&V into pieces and placing it within easy reach of the child. Some parents mentioned that they could increase the intake by offering cut vegetable ‘sticks’ before dinner during the so-called ‘hell hour’ right after school, or pair them with dip sauce and offering it while the children watched television. *’We often cook only the amount that every person will eat*,* we could probably prepare an extra broccoli or a swede turnip or something and it would get eaten. And then maybe make carrot sticks and cucumber sticks before dinner when everybody is a bit hungry and cranky’.—Mother*,* D.*

#### Satisfied with current consumption

At the end of each interview, parents were asked to rate their satisfaction with their family’s intake of F&V on a scale from 1 to 10, where 1 represented ‘very unsatisfied’ and 10 ‘completely satisfied’. Most parents reported being fairly satisfied, with nearly all giving overall ratings between 7 and 10. Some provided separate ratings for fruit and vegetables, often assessing their family’s vegetable intake as being more satisfactory. *’An eight. It has to do with me*,* I would want to have more imagination and make more fun salads and vegetables to go with the dinner sometimes. But it’s not always fun a Thursday in November (laughing).’—Father*,* E.* Half of the interviewees gave the maximum score (10) for either vegetables or fruit—or both. These were, to some extent, the same parents who estimated the recommended daily intake of F&V to be 350 g. *‘Nine. Or should I say ten. I think we eat a lot*,* I do. We have a small fruit and vegetables vendor close to our home*,* we go there*,* she (the child) likes to go by herself this summer and buy some*,* so I do think we are good at it’. — Mother*,* F.* Several parents appeared to evaluate their family’s F&V consumption primarily based on whether vegetables were regularly served with dinner. The inclusion of a salad or other vegetables at the evening meal was often described as satisfactory, while fewer parents referred to F&V consumption across multiple meals and eating occasions throughout the day. *’(Vegetables) It’s every day. Because the salad*,* the green salad is mandatory’.—Father P.* Only two parents rated their family’s intake below 5 in either category, typically referring to a single family member who strongly disliked F&V.

### Theme: parental intentions

Parents described varying intentions when serving F&V to their children, reflecting priorities and beliefs. Four overarching parental intentions were identified in the data: providing nutrients, providing access, providing knowledge and skills, and providing models. One intention focused on ensuring that children consumed F&V in the present moment to obtain immediate nutritional benefits, occasionally considering vitamin supplements when F&V were refused. Another intention was making F&V readily available throughout the day, so that children could choose them freely. A third intention stressed the importance of equipping children with knowledge and practical skills, encouraging them to learn about the health benefits of F&V and to develop practical food preparation skills, such as peeling, chopping, grating, boiling and seasoning. Finally, the fourth intention focused on modelling desired behaviours to influence the children through social and behavioural learning. These four intentions were associated with different strategies for influencing children’s F&V consumption, which are further described and illustrated through the conceptualisation of parental roles.

### Conceptualisation: parental roles when influencing children’s F&V consumption

**Fig. 1:**
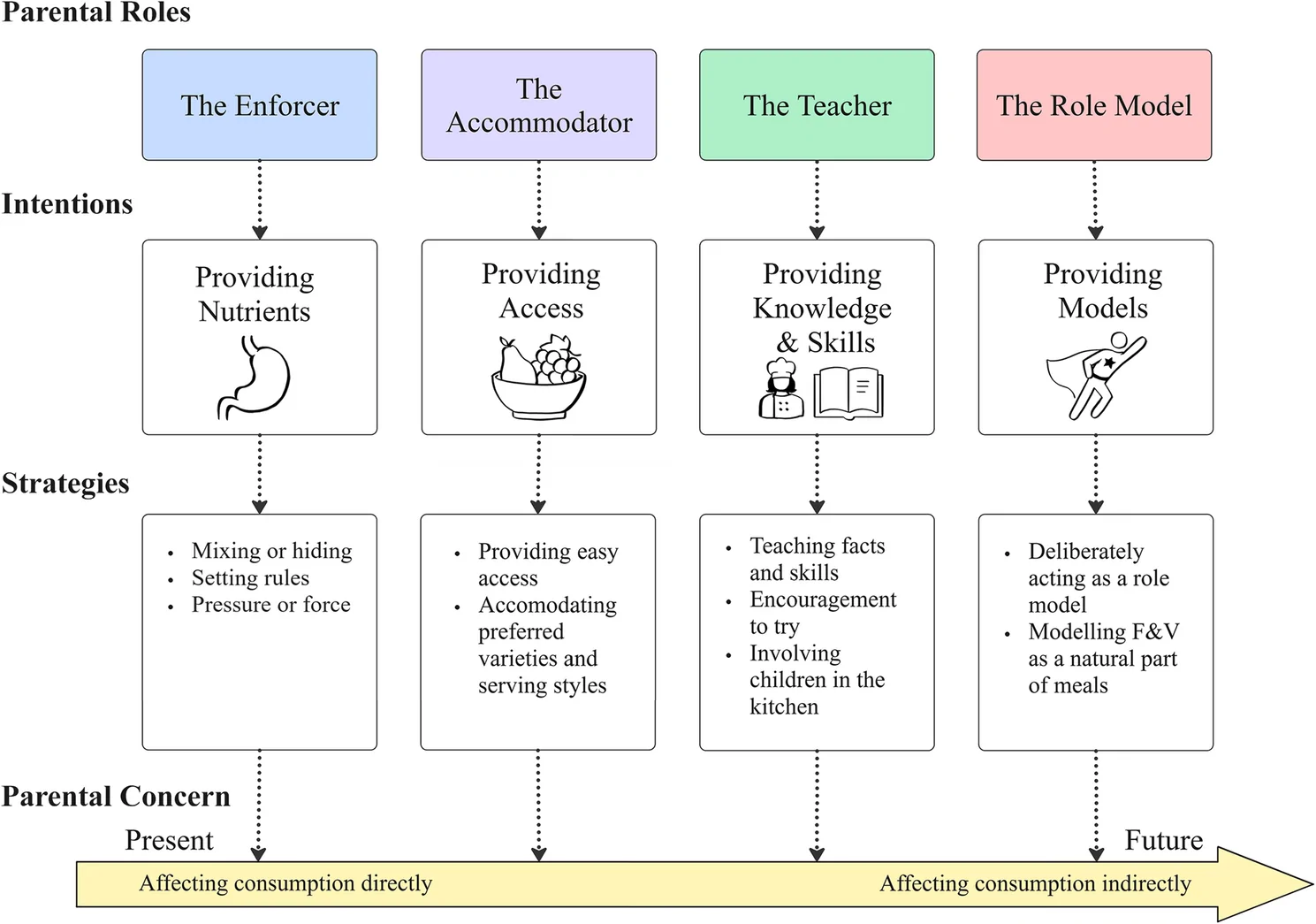
Conceptualised parental roles when influencing 10–12-year-old children’s fruit and vegetable (F&V) consumption. To illustrate patterns in the participating parents’ intentions and strategies related to influencing children’s F&V consumption, four parental roles were conceptualised. The roles are not mutually exclusive and individual parents’ intentions and actions could be described by several roles. The roles range from direct influence on consumption to more indirect and future-oriented approaches

After the thematic analysis, four parental roles when influencing F&V consumption were conceptualised to illustrate patterns observed in parents’ intentions and strategies. Each role originated from a particular parental intention and was associated with two or three strategies used to promote children’s F&V intake (Fig. 1). Individual parents’ actions could be described by several parental roles, with the roles representing analytical conceptualisations rather than mutually exclusive classifications of individual parents. In the following section, we illustrate the various ways the participating parents sought to influence their children’s F&V consumption.

#### The enforcer

The Enforcer role describes intentions and actions directed towards ensuring immediate nutrient intake, often through direct strategies to increase children’s F&V consumption. It was common to mix or hide vegetables in other foods, making the pieces so small and well blended that they were not detected by the child. Some parents would then lie or not provide information about the content of the meal if the child asked about it. A few parents used varying degrees of pressure to get the children to eat F&V. This could involve telling the children they had to eat a particular vegetable the parent considered important or insisting that they finish all vegetables on their plate. Several parents had established rules concerning F&V, especially vegetables at dinnertime. *Strategies: Mixing or hiding F&V in other foods*,* Applying pressure or forcing*,* Setting rules.*

‘I grate carrots and onions finely, so it doesn’t show, they don’t need to know.’ — Mother, O.

‘Smuggling things down, hide things in the bolognese.’ — Mother, G.

‘Avocado must be there every day, it has same substance as mothers milk, I force my children to eat it.’ — Mother, L.

‘With vegetables I don’t let them choose, It’s me who chooses, I know what’s best for him.’ —Father, P.

‘They have to eat one each day… at least once a day.’ — Mother, J.

#### The accommodator

The Accommodator role describes intentions and actions directed towards increasing access to F&V and accommodating children’s preferences. This meant parents making F&V readily available, often at multiple meals or in between meals, placing them where children typically spent time or looked for food when hungry. This contrasted with other families who were satisfied with simply having F&V available in the home, even when kept out of sight. Some parents described cutting vegetables in sticks or smaller pieces and making them available to the children while the parents prepared dinner. This was often aimed primarily at younger siblings but was appreciated also by the older children. Some parents actively asked their children what varieties of F&V they wanted before going grocery shopping or brought the children to the store. Most parents could describe exactly which varieties the children liked and disliked, as well as their preferred serving styles, but the extent to which parents were willing to accommodate these preferences varied. *Strategies: Providing access*,* Accommodating preferred varieties and serving styles.*

‘We always have carrots. If you don’t put it (on the table) no one will eat it.’ — Mother, C.

‘I make a bowl of fruit… It’s nice if we sit in the sofa and watch tv and have (pieces of) fruit with a dip sauce… A trick is you cut it and put a bit of cinnamon on.’—Mother, D.

‘It has always been fruit and vegetables on the table. it’s more natural that you take some. .she likes things laid out nicely… doesn’t like to mix things’ — Mother, N.

‘We make a grocery list, they can provide input. They decide for themselves, you are eating with your eyes, it should look appetising and exciting.’ — Mother, M.

‘“Isn’t dinner ready soon?”, “Sure in 10 minutes, have some vegetables in the meanwhile” and there are already made (carrot and cucumber) sticks they can nibble on.’—Father, E.

#### The teacher

The Teacher role describes intentions and actions directed towards building children’s knowledge, skills, and understanding related to F&V consumption. Some parents taught seasonality, helping children choose F&V appropriate to the time of year. Others linked F&V to health and the body, listening to food-related podcasts or audiobooks together with their children. A few emphasised transparency, explaining nutritional content rather than hiding F&V in meals, thereby challenging the notion that they are something to be disguised. Several parents believed that knowledge could motivate consumption. Encouragement was common, ranging from simple reminders to trying new varieties together. Some grew F&V at home or in relatives’ gardens, aiming to foster curiosity and enjoyment during harvesting. Across these families, there was a shared view that F&V consumption should never be forced. In some households, children regularly helped prepare meals or were given opportunities to be creative with F&V in the kitchen. *Strategies: Teaching facts and skills, Encouragement to try, Involving children in the kitchen.*

‘We heard a podcast comparing gut bacteria to Luke Skywalker and Darth Vader, and that stuck with my child—he now asks if food is good for the Lukes. He sees vegetables as helpful. Teaching can really motivate.’ — Mother, D.

‘A fun thing, in the store we try to find a new vegetable or fruit we have not tried before… we buy it and try it at home, Like—it looks fun, I wonder what it tastes?!’ — Mother, A.

‘Chop, slice, peel carrots… She can fix it all herself.’ — Mother, F.

’Your body needs it. I think you have to explain for them to know it’s important.’—Mother, K.

‘It’s the children job, they fix the vegetables. .make sculptures of fruit, beautiful shapes… The children take part in planting tomato seeds also… it’s cool, not just healthy.’ — Mother, B.

#### The role model

The Role Model role describes intentions and actions directed towards influencing children through parental behaviour, modelling F&V consumption and establishing habits that made F&V a natural part of meals. Several parents referred to the idea that ‘children do what parents do’, with some quoting variations of ‘monkey see, monkey do’. While a few acted as role models unconsciously, others adopted it as a deliberate strategy and could comment on the absence of role modelling in other families they knew. Some parents emphasised early exposure, during infancy, toddlerhood, or even pregnancy, believing that familiarity would lead to acceptance. The long-term aim was to make F&V a consistent and natural part of the diet. *Strategies: Deliberately acting as a role model, Modelling F&V as a natural part of meals.*

‘Lead by example… hard to tell kids to eat broccoli if mum doesn’t.’ — Mother, D.

’Children do what we do. I’m a firm believer of that.’—Mother, C.

‘It’s a natural part of the meal, you get used to it, a human of habits… .’ —Father, H.

‘We chomp down vegetables… kids don’t do as you tell them, but as you do.’ — Mother, B.

‘It’s like a natural part… I think it has to do with what they see us eating, wanting the same.’ —Father, E.

## Discussion

This study explored parents’ perspectives on F&V, their rationale for serving them, and their experiences when influencing their children’s consumption. All parents demonstrated some awareness of the health benefits of F&V, although knowledge of recommendations was generally limited. Despite this, most parents reported being satisfied with their family’s current F&V consumption. The thematic analysis generated five themes, from which patterns in parents’ intentions and strategies were further conceptualised into four parental roles: *The Enforcer*,* The Accommodator*,* The Teacher*, and *The Role Model*.

### Between satisfaction and action—parental awareness of F&V needs

Previous research suggests that parental knowledge of dietary guidelines can influence children’s F&V consumption, particularly among families with higher educational attainment [51]. In the present study participating parents reported being mostly satisfied or very satisfied with their family’s intake of F&V. One interpretation is that these families may consume more F&V than the national median, possibly reflecting a pre-existing interest in the topic, given that they agreed to participate in a study focused on children’s F&V consumption. Alternatively, since many parents appeared unaware of the recommended intake, they may have overestimated how well their family’s consumption aligned with dietary guidelines. Their satisfaction may also reflect that the parents themselves largely define and regulate the level of F&V provision within the family, and in the absence of clear awareness of recommendations, they may not have a specific benchmark against which to evaluate their intake. While most parents felt capable of increasing their children’s F&V intake if needed, and could articulate strategies for doing so, few expressed intentions to act. This may reflect a belief that current intake is sufficient, but also that they have competing priorities, or limited energy to implement changes. Taken together, these findings suggest a potential mismatch between public health messages such as recommendations on why and how much F&V to eat, and parents’ perceptions of F&V, including what constitutes an adequate intake. It highlights a need for more accessible communication of F&V recommendations and their purpose. However, identifying sources of such information that parents feel they can trust is a possible topic for future research.

### Sugar

In previous research on F&V consumption, parents have reported concerns related to the cost of F&V, the short shelf life of produce, and children’s reluctance to eat it, rather than associations between F&V and potential negative health effects [39]. The concerns about sugar expressed by participating parents in the present study could possibly reflect Swedish or Nordic dietary trends in the media during the years preceding the interviews. A qualitative study of Danish and French parents discussing and interacting around the topic of sugar on social media showed that sugar can evoke strong emotions among parents and create uncertainty regarding what is considered sugar [52]. In a study by Wanselius et al. using nationally representative dietary intake data from Swedish adolescents, most free and added sugar intake originated from nutrient-poor food sources such as sweets, sweet bakery products, desserts, sweet dairy products and, in particular, sugar-sweetened beverages [53]. Fruit juice contributed 11% of free sugar intake but 0% of added sugar intake, contrasting with the views expressed by some parents in the present study, who believed that the problem with commercially produced fruit juice was the addition of sugar. These findings highlight the importance of tailoring public health messaging to regional dietary contexts and food cultures. In a Swedish or Nordic context, this may include addressing misconceptions regarding sugar in fruit and fruit- and vegetable-based dishes, to avoid unnecessary restriction of F&V offered to children.

### Parental roles in relation to parenting styles and food parenting practices

Previous literature describes general parenting styles encompassing broader parent–child interactions [54], as well as feeding styles, feeding practices [55] and food parenting practices [56]. Parenting styles, which share terminology with feeding styles, are typically classified according to levels of responsiveness (warmth and emotional support) and demandingness (expectations and behavioural control). A paper by Blissett [55] explored how parenting styles, feeding styles and feeding practices relate to F&V consumption in younger children. The findings suggested that low control, as seen in permissive styles, or minimal use of feeding practices may be associated with lower F&V intake, while excessive control, typical of authoritarian styles, may hinder acceptance. In contrast, authoritative parenting, combining warmth with clear expectations, is considered most beneficial for healthy eating. Similar associations have been reported in several other studies. For example, Patrick et al. [57] studied caregivers of preschool-aged African American and Hispanic children and found that an authoritative parenting style was associated with greater availability of F&V, whereas an authoritarian parenting style was associated with lower reported F&V consumption among young children.

Previous research on feeding and general parenting styles has primarily focused on parents of younger children, who may face age-specific challenges, such as children having tantrums when served vegetables they dislike, which can influence parents’ willingness to offer these foods [40].

For older children, Beltran et al. [43] showed that parents of 10–14-year-olds responded to children’s agency rather than simply overriding it, and general parenting style appeared to have little influence on how parents responded to children’s requests for F&V. Parents listened when children requested specific F&V and allowed them to influence the purchase and availability of F&V when possible. In another study of 4,555 11-year-old children from four European countries, the association between general parenting style and children’s F&V intake was weak [58].

Food parenting practices include both intentional and non-intentional interactions between parents and children that influence children’s behaviours, beliefs, and attitudes about food and eating [56, 59]. The systematic review and meta-analysis of parental autonomy-supportive food parenting practices by Costa Lopes et al. [29] as well as Yee at al. [60] describe age-dependent effects of food parenting practices. The analysis by Costa Lopes showed no significant associations between verbal encouragement or praise and children’s F&V intake among older children (7–12 years), in contrast to the findings for younger children. However, among older children, negotiation (regarding food choices, portion sizes of F&V, and expectations concerning F&V consumption) was associated with higher vegetable consumption in both a cross-sectional and a longitudinal study [35, 36], as was supporting children’s autonomy by providing opportunities for food choices and valuing children’s opinions [61]. Food parenting practices described in the present qualitative study highlight that some participating parents deliberately used several of these strategies, such as supporting autonomy by teaching about F&V and providing children with opportunities and responsibility to practise related skills.

The four conceptualised parental roles included elements similar to general parenting styles but did not map directly onto them. The roles also incorporated some specific food parenting practices used by the participating parents, but situated these practices within the broader intentions they appeared to serve and along a continuum ranging from direct influence on consumption to more indirect and future-oriented influence. Thereby, the conceptualised parental roles may possibly be used as a complement to previous literature when discussing how parents consciously influence F&V consumption among 10–12-year-old children. One potential contribution of the parental roles is that they illustrate feeding practices described by the participating parents in a way that may be easier to communicate and discuss with parents similar to those who participated in the present study. As such, the roles could potentially serve as a starting point for discussions between healthcare professionals and parents. The intentions and strategies represented by the roles could then be discussed in relation to an individual family’s preferences and current feeding practices as well as existing research regarding potentially supportive or, in the case of pressure or force, less supportive food parenting practices.

### Presence in the kitchen and models for eating

Interactions between parents and children influence how both parties engage with F&V—whether through serving, eating, or rejecting them. Parents are affected by their children’s requests [43] and their willingness to try what is served, while children respond to the ways in which parents support their intake. Aspects of this dynamic were evident in a previous study where the children of the parents in the current study were interviewed about their experiences with F&V. The study showed that the children’s engagement with F&V was much impacted by sensory preferences, social context, knowledge, and habitual behaviours—many of these influenced by their parents [45].

The strategy of hiding vegetables in other foods was reported by some parents in the current study, a practice also described in previous literature [39, 62]. In a study by Spill et al. [63] introducing pureed vegetables into other foods (such as tomato sauce with pasta, zucchini bread and chicken casserole) resulted in increased vegetable intake in 3-6-year-olds. Another study by Pescud and Pettigrew [37] found that parents in low-socioeconomic status Australian families with overweight children often concealed vegetables in meals as a coping mechanism to avoid conflict about the vegetables. While this approach increased F&V intake, it also led to children being excluded from the kitchen, as parents feared revealing the hidden ingredients. Not being welcome in the kitchen during meal preparations potentially limits opportunities for children to learn about food preparation and become familiar with F&V. In the present study, intentions and actions represented by *The Enforcer* and The *Teacher* reflected contrasting approaches to children’s involvement in food preparation, with concealment strategies often limiting involvement and teaching-oriented strategies actively encouraging it. Having children present in the kitchen provides a chance to explore flavours, cooking methods, and preparation together. This may lead to what previous literature has described as the ‘IKEA effect’ [64], where active participation increases the perceived value of an activity, making children more willing to taste vegetables they have helped prepare. For children less fond of vegetables, Holley et al. [40] have described a balanced approach as perhaps being beneficial—combining short-term strategies such as mixing F&V into familiar dishes with long-term efforts to expose children to whole F&V, fostering familiarity and eventual acceptance. *The Role Model* role was characterised by efforts to normalise F&V consumption by making it a routine part of every meal, a strategy supported by previous research. For example, Draxten et al. [65] found that when parents model eating F&V at both main meals and snacks, it was associated with children’s adequate intake of F&V. Some participants in the current study also referred to the Swedish Food Agency’s *The Plate Model* [66], a widely recognised guide for balanced eating, communicated to the Swedish public since 1992 (illustrating 1/5 of the plate covered by protein-rich foods, 2/5 of the plate with carbohydrate-rich foods and 2/5 of the plate with F&V). The parents who mentioned *The Plate Model* viewed it as a practical and commonsense tool for ensuring variety and including F&V in daily meals.

### Force or pressure

While some participating parents in the present study reported using pressure or force to make their children eat F&V, previous research suggests that such strategies may be counterproductive. Evidence reviewed by Blissett suggests that encouraging children to try F&V may support acceptance, whereas pressure to eat appears detrimental to acceptance.

However, the associations between pressure and low intake may also reflect broader family eating patterns, as parents who apply pressure tend to consume less F&V themselves and have children with similarly low intake [55]. Practices such as nagging or coercion are likely to reduce children’s acceptance of F&V [67], nevertheless, some parents may feel that without a degree of pressure or negotiation, their children would not consume any F&V at all—particularly given that vegetables are among the least preferred foods for many children [68, 69]. Parental feeding practices are often affected by characteristics of the individual child, including concerns related to weight and eating behaviour [70, 71]. While pressure may negatively affect acceptance, it may also be a response to low acceptance rather than its cause, with parents resorting to such strategies when they perceive their child’s F&V intake to be inadequate. As Blissett [55] notes, even parents with a warm and authoritative general parenting style may resort to coercive practices when particularly concerned about their child’s diet. Importantly, such practices may be less harmful when embedded within a home environment that remains high in warmth and support. In this study, many parents expressed clear expectations regarding F&V intake without resorting to forcing consumption, for example by setting guidelines or benchmarks such as including ‘always two colours of vegetables on the dinner plate,’ signalling structure and intention without applying direct pressure to eat everything on the plate.

### Barriers

Obstacles to F&V consumption have been described in previous research with parents of younger children. A cross-sectional online survey of UK parents of 2–4-year-olds reported common barriers to effective vegetable-feeding practices [72] which included children’s dislike of vegetables, limited parental time, and the influence of extended family. In a US study exploring barriers to healthy eating among school-aged children, parents of 6–12-year-olds cited time and cost as hindrances but also described strategies to overcome them. Additional reported barriers included picky eating, children requesting junk food, early exposure to unhealthy foods, and—among parents of children with overweight—a lack of support from partners or spouses [38]. These findings overlap with the current study, which identified obstacles such as cost, picky eating, preferences for other foods, and differing views within families on how to approach F&V. Notably, only one parent in the present study spontaneously identified lack of time as a significant barrier to serving F&V.

### Methodological considerations, limitations and strengths

Transferability of qualitative findings should always be considered with care. Although the study included parents with diverse cultural backgrounds and was conducted in both a multicultural city and a small Swedish village, the research was situated within a distinctly Swedish social and policy context, including subsidised childcare and healthcare, and free school meals. Parental feeding practices differ across cultural contexts [29] and children’s exposure to fruit and vegetables may unfold differently in countries with other welfare systems, food environments, and cultural norms. Future research may explore if the conceptualised parental roles developed in the present study would be useful to illustrate parents’ intentions and strategies in other geographical locations and cultural contexts.

The use of purposive sampling successfully captured a breadth of experiences, yet participants were not intended to be statistically representative, and those who chose to participate may have had a particular interest in food or parenting. Most parents in this study reported being mostly or very satisfied with their family’s F&V intake, which may reflect a more positive perspective on the topic, or a ‘happy lens’. As parents largely determine which F&V are purchased, served, and made available within the home, this satisfaction may partly reflect their own feeding practices rather than adherence to dietary recommendations. Nevertheless, the interviews contained extensive descriptions of difficulties, including resistance to specific F&V, selective eating, negotiation, costs limiting what could be offered, and parental concerns regarding intake. The findings could therefore be interpreted as reflecting both satisfaction with current practices and ongoing challenges in everyday family food provision.

Educational attainment among participants was generally quite high, with a majority having completed more than 12 years of education (67% in comparison to 58% for the national mean of parents of school-aged children during the year of the data collection) [48]. In several families the co-parent had lower educational attainment, including two cases where the co-parent had nine years of schooling or less; however, it was typically the more highly educated parent who chose to participate in the interviews which is a limitation to the study. A broader representation of parents with a lower educational attainment may have yielded additional insights. Future research would benefit from strategies aimed at improving participation among these parents.

Children of both sexes were evenly represented across participating families. However, parental participation was notably skewed, with twelve mothers and only three fathers volunteering to be interviewed, and no families opting for the possibility of joint interviews. Whether this imbalance reflects gendered norms around food and caregiving in the Swedish or maybe global context, or if other factors also affected is unclear. Nevertheless, it represents a limitation, as mothers and fathers may differ in feeding practices [73] and the paternal perspectives may be underrepresented. It is noteworthy, however, that the fathers who did take part were actively involved in feeding practices and provided rich, detailed accounts.

As with most qualitative studies, a larger or more geographically varied sample might have revealed further nuances. However, a pragmatic approach was applied, and the sample size aligns with comparable qualitative studies, focusing on having a sample that can yield sufficient depth and richness for a meaningful analysis. The purposive sampling was used to achieve relevance and diversity, particularly regarding cultural backgrounds and areas of residence (high, medium, and low socioeconomic areas). The varied perspectives contributed to a substantial empirical foundation, and the data provided sufficient depth and breadth to support the themes and conceptualisation of parental roles in the current context.

Although the study focused on parents of children aged 10–12 years, many participants also had children of other ages (older and younger siblings) and occasionally reflected on experiences from their children’s earlier developmental stages. These accounts enriched the data by offering broader insights into family feeding dynamics over time, but they also extend the scope beyond the intended age group. The findings should therefore be interpreted as reflecting both current practices and more general parental reasoning across childhood.

A strength of the present study is the cultural diversity and the varied family structures represented in the sample. Another strength lies in the openness and earnestness with which parents shared their thoughts and experiences. This was facilitated by an intentionally warm, curious, and non-judgemental interview environment, something several participants noted as very supportive.

By capturing the participating parents’ reasoning, experiences and knowledge underpinning their feeding practices, this qualitative study extends the existing literature, illuminating examples of both the strategies parents describe and the concerns in which these strategies exist. The study contributes insights from a relatively understudied group, namely parents of older children, and from a Swedish/Nordic context. Findings relating to limited awareness of F&V recommendations, concerns about sugar in fruit and vegetable dishes, and the conceptualisation of parental roles provide additional perspectives on how these parents reason about and seek to influence F&V consumption during late childhood.

## Conclusion

In conclusion, the experiences and beliefs expressed by the participating parents suggest several barriers to supporting adequate F&V intake among their 10–12-year-old children. These included limited awareness of recommendations and many parents not recognising how everyday practices, such as offering F&V at multiple meals throughout the day, may contribute to achieving recommended intake levels. The limited awareness of recommendations may make it more difficult for parents to translate their intentions regarding F&V into practices that support recommended intake levels. Additionally, concerns about sugar content sometimes led to unnecessary restriction of F&V, which may be relevant to consider when planning interventions with similar groups of parents. When considered alongside previous research, the findings of this study suggest that providing more accessible guidance not only on *why*, but also on *how*,* how much*, and *how often* to serve F&V may support parents similar to those included in the present study. The four conceptualised, non-mutually exclusive parental roles, *The Enforcer*,* The Accommodator*,* The Teacher*, and *The Role Model*, describe patterns identified in the data and illustrate one way of considering the participating parents’ intentions and strategies related to F&V. The roles may offer a starting point for discussions between healthcare professionals and parents similar to those included in the present study regarding different ways of supporting children’s F&V intake. Such discussions may provide opportunities to consider additional strategies alongside the parents’ existing food parenting practices.

## Supplementary Information

Supplementary Material 1.

Supplementary Material 2.

## Data Availability

The dataset generated and analysed during the current study is not publicly available due to the conditions in the ethical approval and the need to protect participants’ privacy. It is available from the corresponding author Hannah Helgegren (hannah.helgegren@gu.se) upon reasonable request.

## Abbreviation

F&V: Fruit and Vegetables

## Declarations

### Declaration of AI-assisted technologies in the writing process

During the preparation of this work the authors used Co-Pilot (with an enterprise data protection licence from the University of Gothenburg) in order to improve language and readability of the manuscript. After using this tool, the authors carefully reviewed and edited the content as needed and takes full responsibility for the content of the published article.

## References

[1] Holsten JE, Deatrick JA, Kumanyika S, Pinto-Martin J, Compher CW. Children’s food choice process in the home environment. A qualitative descriptive study. Appetite. 2012;58(1):64–73. 10.1016/j.appet.2011.09.002 21986183

[2] Norman Å, Nyberg G, Elinder LS, Berlin A. Parental strategies for influencing the diet of their children – A qualitative study from disadvantaged areas. Appetite. 2018;125:502–11. 10.1016/j.appet.2018.03.002 29524473

[3] Swedish Food Agency. Riksmaten barn (National Survey of Childrens’ Food Habits). 2003.

[4] Rashid V, Engberink MF, van Eijsden M, Nicolaou M, Dekker LH, Verhoeff AP, et al. Ethnicity and socioeconomic status are related to dietary patterns at age 5 in the Amsterdam born children and their development (ABCD) cohort. BMC Public Health. 2018;18(1):115. 10.1186/s12889-017-5014-0 PMC5759294 29310648

[5] Serasinghe N, Vepsäläinen H, Lehto R, Abdollahi AM, Erkkola M, Roos E, et al. Associations between socioeconomic status, home food availability, parental role-modeling, and children’s fruit and vegetable consumption: a mediation analysis. BMC Public Health. 2023;23(1):1037. 10.1186/s12889-023-15879-2 PMC10233887 37259139

[6] Gautam N, Rahman MM, Khanam R. Socioeconomic inequalities in health behaviors in children and adolescents: evidence from an Australian cohort. BMC Public Health. 2025;25(1):314. 10.1186/s12889-025-21472-6 PMC11762062 39856619

[7] Pearce A, Dundas R, Whitehead M, Taylor-Robinson D. Pathways to inequalities in child health. Arch Dis Child. 2019;104(10):998–1003. 10.1136/archdischild-2018-314808 PMC6889761 30798258

[8] WHO. Healthy Diet 2020 [Available from: https://www.who.int/news-room/fact-sheets/detail/healthy-diet.

[9] European Commission. Food based dietary guidelines Europe, Knowledge-4-policy-EU 2025 [updated July 21. 2025. Summary of food based dietary guidelines from all European countries]. Available from: https://knowledge4policy.ec.europa.eu/health-promotion-knowledge-gateway/food-based-dietary-guidelines-europe-table-3_en.

[10] Wang DD, Li Y, Bhupathiraju SN, Rosner BA, Sun Q, Giovannucci EL, et al. Fruit and Vegetable Intake and Mortality: Results From 2 Prospective Cohort Studies of US Men and Women and a Meta-Analysis of 26 Cohort Studies. Circulation. 2021;143(17):1642–54. 10.1161/CIRCULATIONAHA.120.048996 PMC8084888 33641343

[11] Aune D, Giovannucci E, Boffetta P, Fadnes LT, Keum N, Norat T, et al. Fruit and vegetable intake and the risk of cardiovascular disease, total cancer and all-cause mortality—a systematic review and dose-response meta-analysis of prospective studies. Int J Epidemiol. 2017;46(3):1029–56. 10.1093/ije/dyw319 PMC5837313 28338764

[12] Collaborators GBDD. Health effects of dietary risks in 195 countries, 1990–2017: a systematic analysis for the Global Burden of Disease Study 2017. Lancet. 2019;393(10184):1958–72. 10.1016/S0140-6736(19)30041-8 PMC6899507 30954305

[13] Wu Y, Zhang D, Jiang X, Jiang W. Fruit and vegetable consumption and risk of type 2 diabetes mellitus: A dose-response meta-analysis of prospective cohort studies. Nutr Metabolism Cardiovasc Dis. 2015;25(2):140–7. 10.1016/j.numecd.2014.10.004 25456152

[14] Liberali R, Kupek E, Assis MAA. Dietary Patterns and Childhood Obesity Risk: A Systematic Review. Child Obes. 2020;16(2):70–85. 10.1089/chi.2019.0059 31742427

[15] Epstein LH, Gordy CC, Raynor HA, Beddome M, Kilanowski CK, Paluch R. Increasing fruit and vegetable intake and decreasing fat and sugar intake in families at risk for childhood obesity. Obes Res. 2001;9(3):171–8. 10.1038/oby.2001.18 11323442

[16] Wang T, Heianza Y, Sun D, Zheng Y, Huang T, Ma W, et al. Improving fruit and vegetable intake attenuates the genetic association with long-term weight gain. Am J Clin Nutr. 2019;110(3):759–68. 10.1093/ajcn/nqz136 PMC6736184 31301130

[17] WHO. Report of the Commission on Ending Childhood Obesity: implementation plan: executive summary. Geneva: World Health Organization; 2017. Contract No.:WHO/NMH/PND/ECHO/17.1.

[18] Mikkilä V, Räsänen L, Raitakari OT, Pietinen P, Viikari J. Consistent dietary patterns identified from childhood to adulthood: The Cardiovascular Risk in Young Finns Study. Br J Nutr. 2005;93(6):923–31. 10.1079/bjn20051418 16022763

[19] Demory-Luce D, Morales M, Nicklas T, Baranowski T, Zakeri I, Berenson G. Changes in food group consumption patterns from childhood to young adulthood: the Bogalusa Heart Study. J Am Diet Assoc. 2004;104(11):1684–91. 10.1016/j.jada.2004.07.026 15499355

[20] Kelder SH, Perry CL, Klepp KI, Lytle LL. Longitudinal tracking of adolescent smoking, physical activity, and food choice behaviors. Am J Public Health. 1994;84(7):1121–6. 10.2105/ajph.84.7.1121 PMC1614729 8017536

[21] Lynch C, Kristjansdottir AG, te Velde SJ, Lien N, Roos E, Thorsdottir I, et al. Fruit and vegetable consumption in a sample of 11-year-old children in ten European countries – the PRO GREENS cross-sectional survey. Public Health Nutr. 2014;17(11):2436–44. 10.1017/S1368980014001347 PMC10282338 25023091

[22] Rosi A, Paolella G, Biasini B, Scazzina F. Dietary habits of adolescents living in North America, Europe or Oceania: A review on fruit, vegetable and legume consumption, sodium intake, and adherence to the Mediterranean Diet. Nutr Metab Cardiovasc Dis. 2019;29(6):544–60. 10.1016/j.numecd.2019.03.003 31078365

[23] Stea TH, Nordheim O, Bere E, Stornes P, Eikemo TA. Fruit and vegetable consumption in Europe according to gender, educational attainment and regional affiliation-A cross-sectional study in 21 European countries. PLoS ONE. 2020;15(5):e0232521. 10.1371/journal.pone.0232521 PMC7219700 32401798

[24] Swedish Food Agency. Riksmaten ungdom (National Survey of Adolescents’ Food Habits). 2018.

[25] Olsson C, Hörnell A, Waling M. High availability of vegetables and fruit through government-funded school lunch is not reflected in 4th grade pupils’ intake. Food Nutr Res. 2023;67:9405. 10.29219/fnr.v67.9405. PMC10392862 37533447

[26] Blanchette L, Brug J. Determinants of fruit and vegetable consumption among 6-12-year-old children and effective interventions to increase consumption. J Hum Nutr Dietetics. 2005;18(6):431–43. 10.1111/j.1365-277X.2005.00648.x 16351702

[27] Braune T, Adams J, Winpenny EM. Exploring the changing association between parental and adolescent fruit and vegetable intakes, from age 10 to 30 years. Int J Behav Nutr Phys Activity. 2024;21(1):56. 10.1186/s12966-024-01604-8 PMC11083755 38730502

[28] Pearson N, Biddle SJ, Gorely T. Family correlates of fruit and vegetable consumption in children and adolescents: a systematic review. Public Health Nutr. 2009;12(2):267–83. 10.1017/S1368980008002589 18559129

[29] Costa Lopes E, Rodrigues Vilella P, Ruffoni Moreira P, Warkentin S, Siqueira Guedes Coelho A, de Mercia G, et al. Parental autonomy-supportive food practices and fruit and vegetable consumption in children: A systematic review and meta-analysis. Appetite. 2026;219:108389. 10.1016/j.appet.2025.108389 41317875

[30] Holley CE, Haycraft E, Farrow C. Why don’t you try it again?’ A comparison of parent led, home based interventions aimed at increasing children’s consumption of a disliked vegetable. Appetite. 2015;87:215–22. 10.1016/j.appet.2014.12.216 25555540

[31] Blissett J, Bennett C, Donohoe J, Rogers S, Higgs S. Predicting successful introduction of novel fruit to preschool children. J Acad Nutr Diet. 2012;112(12):1959–67. 10.1016/j.jand.2012.08.014 23174683

[32] Wardle J, Cooke LJ, Gibson EL, Sapochnik M, Sheiham A, Lawson M. Increasing children’s acceptance of vegetables; a randomized trial of parent-led exposure. Appetite. 2003;40(2):155–62. 10.1016/s0195-6663(02)00135-6 12781165

[33] Wardle J, Herrera ML, Cooke L, Gibson EL. Modifying children’s food preferences: the effects of exposure and reward on acceptance of an unfamiliar vegetable. Eur J Clin Nutr. 2003;57(2):341–8. 10.1038/sj.ejcn.1601541 12571670

[34] Holley CE, Farrow C, Haycraft E. A Systematic Review of Methods for Increasing Vegetable Consumption in Early Childhood. Curr Nutr Rep. 2017;6(2):157–70. 10.1007/s13668-017-0202-1 PMC5438436 28596931

[35] Vereecken C, Legiest E, De Bourdeaudhuij I, Maes L. Associations between general parenting styles and specific food-related parenting practices and children’s food consumption. Am J Health Promot. 2009;23(4):233–40. 10.4278/ajhp.07061355 19288844

[36] Vereecken C, Haerens L, De Bourdeaudhuij I, Maes L. The relationship between children’s home food environment and dietary patterns in childhood and adolescence. Public Health Nutr. 2010;13(10A):1729–35. 10.1017/S1368980010002296 20883573

[37] Pescud M, Pettigrew S. Parents’ experiences with hiding vegetables as a strategy for improving children’s diets. Br Food J. 2014;116(12):1853–63.

[38] Nepper MJ, Chai W. Parents’ barriers and strategies to promote healthy eating among school-age children. Appetite. 2016;103:157–64. 10.1016/j.appet.2016.04.012 27090341

[39] Nepper MJ, Chai W. Parental Views of Promoting Fruit and Vegetable Intake Among Overweight Preschoolers and School-Aged Children. Global Qualitative Nurs Res. 2017;4:2333393617692085. 10.1177/2333393617692085 PMC5305122 28462357

[40] Holley CE, Farrow C, Haycraft E. Investigating offering of vegetables by caregivers of preschool age children. Child Care Health Dev. 2017;43(2):240–9. 10.1111/cch.12400 27554997

[41] Porter L, Chater AM, Haycraft E, Farrow C, Holley CE. Role-model, reoffer, reward: A thematic analysis and TDF mapping of influences on families’ use of evidence-based vegetable feeding practices. Appetite. 2023;189:106764. 10.1016/j.appet.2023.106764 37442525

[42] George L, Davison J. Using the Behaviour Change Wheel and Theoretical Domains Framework to identify factors related to increasing fruit and vegetable consumption in children to guide future intervention development. Public Health Nutr. 2024;27(1):e253. 10.1017/S1368980024002362 PMC11705016 39632715

[43] Beltran A, O’Connor TM, Hughes SO, Thompson D, Baranowski J, Nicklas TA, et al. Parents’ Qualitative Perspectives on Child Asking for Fruit and Vegetables. Nutrients. 2017;9(6):575. 10.3390/nu9060575. PMC5490554 28587236

[44] Braun V, Clarke V. Using thematic analysis in psychology. Qualitative Res Psychol. 2006;3(2):77–101.

[45] Helgegren H, Winkvist A, Lepp M, Larsson C. There are healthy things inside’: Children’s thoughts about, experiences with and approaches to vegetable and fruit consumption. Appetite. 2025;206:107819. 10.1016/j.appet.2024.107819 39667597

[46] Swedish National Agency for Education. Elevstatistik grundskolan Malmö (Statistics concerning Students in grade 1–9, in Malmö). 2017.

[47] Swedish National Agency for Education. Elevstatistik grundskolan Staffanstorp (Statistics concerning Students in grade 1–9, in Staffanstorp). 2017.

[48] Swedish National Agency for Education. Elevstatistik grundskolan Riket (Statistics concerning Students in grade 1–9 in Sweden). 2017.

[49] Pescud M, Pettigrew S, Wood L, Henley N. Insights and recommendations for recruitment and retention of low socio-economic parents with overweight children. Int J Soc Res Methodol. 2015;18(6):617–33.

[50] Berger PL, Luckmann T. The Social Construction of Reality: A Treatise in the Sociology of Knowledge. Anchor; 1967.

[51] Lehto E, Ray C, Te Velde S, Petrova S, Duleva V, Krawinkel M, et al. Mediation of parental educational level on fruit and vegetable intake among schoolchildren in ten European countries. Public Health Nutr. 2015;18(1):89–99. 10.1017/S136898001300339X PMC10271320 24476635

[52] Moura AF, Aschemann-Witzel J. Perspectives on sugar consumption expressed on social media by French-speaking and Danish-speaking parents. Soc Sci Med. 2021;270:113636. 10.1016/j.socscimed.2020.113636 33433373

[53] Wanselius J, Lindroos AK, Moraeus L, Patterson E, Berg C, Larsson C. Dietary sources of free, added, and total sugars in Swedish adolescents. Eur J Nutr. 2024;64(1):57. 10.1007/s00394-024-03568-8 PMC11685256 39738881

[54] Darling N, Steinberg L. Parenting style as context: An integrative model. American Psychological Association; 1993. pp. 487–96.

[55] Blissett J. Relationships between parenting style, feeding style and feeding practices and fruit and vegetable consumption in early childhood. Appetite. 2011;57(3):826–31. 10.1016/j.appet.2011.05.318 21651932

[56] Vaughn AE, Ward DS, Fisher JO, Faith MS, Hughes SO, Kremers SP, et al. Fundamental constructs in food parenting practices: a content map to guide future research. Nutr Rev. 2016;74(2):98–117. 10.1093/nutrit/nuv061 PMC4892304 26724487

[57] Patrick H, Nicklas TA, Hughes SO, Morales M. The benefits of authoritative feeding style: caregiver feeding styles and children’s food consumption patterns. Appetite. 2005;44(2):243–9. 10.1016/j.appet.2002.07.001 15808898

[58] De Bourdeaudhuij I, Te Velde SJ, Maes L, Perez-Rodrigo C, de Almeida MD, Brug J. General parenting styles are not strongly associated with fruit and vegetable intake and social-environmental correlates among 11-year-old children in four countries in Europe. Public Health Nutr. 2009;12(2):259–66. 10.1017/S1368980008002930 18616848

[59] O’Connor TM, Mâsse LC, Tu AW, Watts AW, Hughes SO, Beauchamp MR, et al. Food parenting practices for 5 to 12 year old children: a concept map analysis of parenting and nutrition experts input. Int J Behav Nutr Phys Act. 2017;14(1):122. 10.1186/s12966-017-0572-1 PMC5594481 28893273

[60] Yee AZ, Lwin MO, Ho SS. The influence of parental practices on child promotive and preventive food consumption behaviors: a systematic review and meta-analysis. Int J Behav Nutr Phys Act. 2017;14(1):47. 10.1186/s12966-017-0501-3 PMC5387370 28399881

[61] Ha AS, Zeng T, Wang L, Ng JYY. Parental Support, Children’s Physical Activity, Dietary Behaviors and Health-Related Quality of Life: Evidence From Three Asian Cities. Int J Behav Med. 2022;29(6):752–61. 10.1007/s12529-022-10056-y 35316508

[62] Peters J, Parletta N, Lynch J, Campbell K. A comparison of parental views of their pre-school children’s ‘healthy’ versus ‘unhealthy’ diets. A qualitative study. Appetite. 2014;76:129–36. 10.1016/j.appet.2014.02.001 24524974

[63] Spill MK, Birch LL, Roe LS, Rolls BJ. Hiding vegetables to reduce energy density: an effective strategy to increase children’s vegetable intake and reduce energy intake. Am J Clin Nutr. 2011;94(3):735–41. 10.3945/ajcn.111.015206 PMC3155937 21775554

[64] Radtke T, Liszewska N, Horodyska K, Boberska M, Schenkel K, Luszczynska A. Cooking together: The IKEA effect on family vegetable intake. Br J Health Psychol. 2019;24(4):896–912. 10.1111/bjhp.12385 31557370

[65] Draxten M, Fulkerson JA, Friend S, Flattum CF, Schow R. Parental role modeling of fruits and vegetables at meals and snacks is associated with children’s adequate consumption. Appetite. 2014;78:1–7. 10.1016/j.appet.2014.02.017 PMC4034448 24630934

[66] Swedish Food Agency. Tallriksmodellen (The Plate Model) 2016 [cited 2024. Available from: https://www.livsmedelsverket.se/matvanor-halsa--miljo/kostrad/tallriksmodellen.

[67] Galloway AT, Fiorito LM, Francis LA, Birch LL. Finish your soup’: Counterproductive effects of pressuring children to eat on intake and affect. Appetite. 2006;46(3):318–23. 10.1016/j.appet.2006.01.019 PMC2604806 16626838

[68] Cooke LJ, Wardle J. Age and gender differences in children’s food preferences. Br J Nutr. 2005;93(5):741–6. 10.1079/bjn20051389 15975175

[69] Ragelienė T. Do children favor snacks and dislike vegetables? Exploring children’s food preferences using drawing as a projective technique. A cross-cultural study. Appetite. 2021;165:105276. 10.1016/j.appet.2021.105276 33971287

[70] Loth KA, Mohamed N, Trofholz A, Tate A, Berge JM. Associations between parental perception of- and concern about-child weight and use of specific food-related parenting practices. Appetite. 2021;160:105068. 10.1016/j.appet.2020.105068 PMC7879703 33352291

[71] Edwards KL, Pickard A, Farrow C, Haycraft E, Herle M, Llewellyn C, et al. Differences in parental behaviour, emotions, and cognitions between children’s eating profiles. Appetite. 2024;202:107641. 10.1016/j.appet.2024.107641 39173839

[72] Arthur S, Mitchell AE, Morawska A. Parent-reported barriers to establishing a healthy diet with young children in Australia. Child Care Health Dev. 2023;49(4):669–78. 10.1111/cch.13081 36394570

[73] Blissett J, Meyer C, Haycraft E. Maternal and paternal controlling feeding practices with male and female children. Appetite. 2006;47(2):212–9. 10.1016/j.appet.2006.04.002 16735080

