# Supplementary material for: Parents’ perspectives and roles in influencing 10–12-year-old children’s fruit and vegetable consumption: a qualitative study in Sweden
Source: BMC Public Health. 2026 Oct 13;26:2953. doi: 10.1186/s12889-026-29852-2 (PMC13660306; doi:10.1186/s12889-026-29852-2)
Supplement: Supplementary file 1 [file 12889_2026_29852_MOESM1_ESM.docx]

**Appendix A** (English Translation)

**Date:** ___________________________
**Study ID:** ________________________

**For parents/legal guardians participating in the High Five Skåne study**

We would like to know a little more about your family. Please answer the questions below. Write your answers and tick or circle the option that fits best where choices are given.
If you have any questions, the study contact person will be happy to help you.

**Who lives with your child?**

*(for example: parent 1, parent 2, siblings born in 2005 and 2001)*

**Which district do you live in?**

*(If you do not know, please write your address)*

**Which school does your child attend?**

**Was your child born in** *(tick or circle one option)*

Sweden

A Nordic country other than Sweden
A European country outside the Nordic countries
A country outside Europe

**Which language(s) does your child speak at home?**

**Questions about you**

State your relationship to the child *(e.g. mother)* **__________________________**

**Which country where you born in?**

**What education do you have?**

*(tick or circle the highest level you have completed)*

No schooling or less than 9 years
Compulsory school (9 years)
Upper secondary school
University/college

**What is your main activity at the moment?**

*(tick or circle the option that fits best)*

Working full-time
Working part-time
Unemployed
On sick leave
Studying
On parental leave
Retired
Other

**Approximately what is your yearly income?** (individual or as a family)

*(tick or circle the option that fits best; answers in Swedish kronor)*

Less than 100,000
100,000 – 200,000
200,000 – 300,000
300,000 – 400,000
More than 400,000
Receiving social welfare
Do not know

**Questions about other parents/legal guardians** (if applicable)

State your relationship to the child *(e.g. father)* **__________________________**

**Which country where you born in?**

**What education do you have?**

*(tick or circle the highest level completed)*

No schooling or less than 9 years
Compulsory school (9 years)
Upper secondary school
University/college

**What is your main activity at the moment?**

*(tick or circle the option that fits best)*

Working full-time
Working part-time
Unemployed
On sick leave
Studying
On parental leave
Retired
Other

**Approximately what is your yearly income?** (individual or as a family)

*(tick or circle the option that fits best; answers in Swedish kronor)*

Less than 100,000
100,000 – 200,000
200,000 – 300,000
300,000 – 400,000
More than 400,000
Receiving social welfare
Do not know
