# Supplementary material for: Parents’ perspectives and roles in influencing 10–12-year-old children’s fruit and vegetable consumption: a qualitative study in Sweden
Source: BMC Public Health. 2026 Oct 13;26:2953. doi: 10.1186/s12889-026-29852-2 (PMC13660306; doi:10.1186/s12889-026-29852-2)
Supplement: Supplementary file 2 [file 12889_2026_29852_MOESM2_ESM.docx]

**Appendix B.**

Supplementary material for ‘Parents’ perspectives and roles in influencing 10–12-year-old children’s fruit and vegetable consumption: A qualitative study in Sweden’.

H. Helgegren, A. Winkvist, C. Larsson

**Semi-structured interview guide for individual parent interviews**

This interview guide was developed for this specific study and was used together with prompting questions (such as: tell me more, explain more, can you say a bit more about that?) The interview guide that was used was in Swedish and is here presented translated to English.

Abbreviations: F&V = fruit and vegetables

**Introduction**

*Start recording. Explain the purpose and structure of the conversation, repeat voluntary participation, and anonymity.*

We will be talking about both fruit and vegetables (F&V). (Show two A4 sized papers with “fruit” and “vegetables” written on them).

Which would you like to start with? *(proceed with the one chosen, let the paper with the word be visible on the table).*

What comes to mind when you hear the word ________? (fruit or vegetables)

What are your thoughts about it? (ask to elaborate further if necessary).

*Then change paper on the table and ask the same question about _________ (fruit or vegetables, the one not chosen first).*

**Own Preferences**

(introduce picture material with F&V photos, keep visible during rest of interview).

What do you personally think about eating vegetables? Which are your favourites? How do you prefer them served? Are there any you don’t like? Any you find intimidating or off-putting? Which vegetables do you usually have at home? Which of these do you eat?

*(Then ask the same question, but about fruit)*

**Child’s Preferences** *(change order of fruit/vegetable depending on first question)*

Are there any vegetables your child likes? Any others?

How does your child prefer their vegetables to be served or prepared?

Can your child prepare any vegetables themselves? Which ones, and how?

Do you notice any differences in what kinds of vegetables your child likes or accepts at home, at school, or when visiting friends or relatives?

Are there any fruits or berries your child enjoys? Any others?

How does your child prefer to eat these? (Cold/room temperature, peeled, sliced etc.?)

**Perceived Benefits**

Do you think children should eat F&V? Why?

*(Explore narratives)*

Do you think adults should eat F&V? Why?

**Influences**

Who taught you what you know about F&V? *(Explore further)*

Who do you feel provides opinions, tips, or advice about F&V? *(Explore further)*

Have you heard anyone say that it’s not good to eat F&V or certain types of them? Who, and what did they say?

*(Explore further:‘*What do you think about that? Does that affect what you choose to do?’*)*

**Strategies**

When do you think your child is at their best when it comes to eating F&V at home?

Did you do anything specific or intentional in those situations?

Do you have any special tricks to get your child to eat the food you serve? To eat F&V?

Can you recall a time when you tried something or were in a specific situation where it worked particularly well for your child to eat fruit or vegetables?

Can you think of any situation where it worked less well?

Do you remember a time when your child surprised you by eating a vegetable or fruit (or other food) you didn’t think they liked?

How many days per week do you think vegetables are served in your home?

Does your child ever help themselves to vegetables?

Where is the fruit kept in your home? *(e.g. in the fridge, fruit bowl on table, other)*

Do you have any rules about how many fruits one must or may eat per day?

Who decides which F&V are bought? Who does the shopping?

**Conditions**

What do you think influences whether children eat more or less F&V?

**Your Family**

How satisfied are you with the amount of F&V your family eats? (*Scale: 1 to 10, where 1 means not at all satisfied and 10 means completely satisfied. Explore further.)*

Is there more than one child in the family? In that case, do you think the siblings’ intake of F&V differs?

Do you feel that you can influence how much F&V is eaten in your home?

If you wanted your children to eat more (or less) F&V, what do you think you could do?

What are your thoughts on fruit juice in your family? (And do the children drink it regularly?)

**Other**

Do you know the recommended daily intake of F&V for children and adults? (Explore further).

Is there anything else about F&V that has come to mind during our conversation? Anything you’d like to elaborate on?

Why did you choose to start our conversation with Fruit/vegetables? *(the one they chose).*

**Closing**

Thank the participant for their generosity with their time and responses. *Stop recording after the participant has left.*
